# Affi-BAMS™: A Robust Targeted Proteomics Microarray Platform to Measure Histone Post-Translational Modifications

**DOI:** 10.3390/ijms241210060

**Published:** 2023-06-13

**Authors:** Ghaith M. Hamza, Eric Miele, Don M. Wojchowski, Paul Toran, Camilla R. Worsfold, Tamil S. Anthonymuthu, Vladislav B. Bergo, Andrew X. Zhang, Jeffrey C. Silva

**Affiliations:** 1 Discovery Biology, Discovery Sciences, R&D, AstraZeneca, Boston, MA 02451, USA; 2 Molecular, Cellular and Biomedical Sciences, University of New Hampshire, Durham, NH 03824, USA; 3 Adeptrix Corporation, Beverly, MA 01915, USA; 4 Cell Signaling Technology, Danvers, MA 01915, USA

**Keywords:** proteomics, histones, PTMs, immunoaffinity peptide capture, MALDI MS, BAMS

## Abstract

For targeted protein panels, the ability to specifically assay post-translational modifications (PTMs) in a quantitative, sensitive, and straightforward manner would substantially advance biological and pharmacological studies. The present study highlights the effectiveness of the Affi-BAMS™ epitope-directed affinity bead capture/MALDI MS platform for quantitatively defining complex PTM marks of H3 and H4 histones. Using H3 and H4 histone peptides and isotopically labelled derivatives, this affinity bead and MALDI MS platform achieves a range of >3 orders of magnitude with a technical precision CV of <5%. Using nuclear cellular lysates, Affi-BAMS PTM-peptide capture resolves heterogeneous histone N-terminal PTMs with as little as 100 µg of starting material. In an HDAC inhibitor and MCF7 cell line model, the ability to monitor dynamic histone H3 acetylation and methylation events is further demonstrated (including SILAC quantification). Affi-BAMS (and its capacity for the multiplexing of samples and target PTM-proteins) thus provides a uniquely efficient and effective approach for analyzing dynamic epigenetic histone marks, which is critical for the regulation of chromatin structure and gene expression.

## 1. Introduction

The proteome and its diverse functions are regulated in highly dynamic ways via post-translational modifications (PTMs) with a diversity beyond that of genomes, transcriptomes, and total protein expression. Among the 400 PTM types, phosphorylation, methylation, acetylation, and ubiquitination are among the most frequent [1]. For phosphorylation, examples include phospho S-, T-, and Y-sites that rapidly modulate cell signal transduction factors, and signaling networks [2,3,4]. Protein Arginine Methyltransferases (PRMT) and Protein Lysine Methyltransferases (PKMT) [5] mediate the methylation of diverse targets, including laminins, ribosomal proteins, PARPs, select protein kinases, and histones [6]. Acetylation events typically occur at target protein N-termini and at K-residues and are mediated by N-and K-acetyltransferases [7,8]. Examples include DNA binding domain acetylation of p53 at K164 and K120 [9], and alpha-tubulin stabilization via K40 acetylation [10]. Ubiquitination is commonly K-Ub (but also M- and S-/T-). This can be ubiquitin chains (poly-), which modulate protein turnover, or single chains (mono-), which more frequently modulate protein function (or trafficking) [11,12,13]. One well-studied example is the DNA-damage-induced mono-ubiquitination of *PCNA* (Proliferating Cell Nuclear Antigen) and its resulting repair activity via DNA polymerase recruitment [14]. More recently, K-Ub has been extensively studied in the realm of proteolysis-targeting chimera (PROTACs), which hijacks the intercellular protein destruction mechanism to degrade proteins of interest [15].

In the context of regulated chromatin structure and gene expression, the histones H2A, H2B, H3, and H4 as DNA-bound nuclear octamers are dynamic and functionally consequential major targets of T-, S-, and Y-phosphorylation, K- and R- methylation, K-acetylation, and K-ubiquitination. These modifications and their combinations are summarized in Supplemental Figure S1. These histone PTM dynamics can also regulate and coordinate cell signaling networks that impact gene expression [16,17,18,19]. This vast complexity is regulated in fluid ways by two main classes of enzymes: writers and erasers. Writers function by adding histone modifications and include HATs and HMTs. Erasers are proteins that remove such PTM modifications from histones and include HKMs and HDACs [20].

Mass spectrometry (MS) is a highly accurate and precise way of measuring the fundamental properties of the mass to charge (*m/z*) of analytes, but it is complicated by the analyte complexity, which can also involve the saturation of inherent dynamic ranges. Sensitivity and sequencing speed thresholds are also often a limiting factor in producing a complete inventory of the proteome of interest. In liquid chromatography mass spectrometry (LC-MS), LC is a powerful technique that is often employed to simplify sample complexities through sorbent interactions. For LC, however, a need exists to generate sufficiently tight chromatographic peaks for signals in MS spectra to be resolved and detected for analytes of interest. For LC, a bottleneck also exists for analyses of multiple samples, and replicates. LC-MS has been widely used to provide high-resolution measurements for analytes of interest [21,22]. Goals to analyze the entirety of proteomes have led to new technologies that push MS boundaries [23,24]. Bottom-up LC-MS data are often acquired through either Data Dependent Acquisition (DDA) or Data Independent Acquisition (DIA) and analyzed via a spectrum-centric or peptide-centric approach, such that a FASTA database or peptide library is utilized to match MS/MS fragmentation data [25,26]. For Matrix Assisted Laser Desorption Ionization (MALDI), a simpler and less costly MS system, certain gains in applications and sensitivity also have been made. For targeted proteomics applications, studies by Borchers and colleagues have enabled the use of immune-MALDI (iMALDI) by combining the immune-affinity enrichment of peptides followed by MALDI MS analysis as a screening method for select markers [27,28,29,30,31]. Likewise, instruments that employ fast acquisition rates and low mass errors have also come to fruition, such as the rapifleX and timsTOF Flex instrument, which display an accuracy of <10 PPM mass error. Such gains make MALDI MS an attractive analytical readout for the Affi-BAMS platform.

Monitoring PTMs on proteins of interest, such as the case of histone modifications, can be often met with significant hurdles. In the case of low-abundance modifications, such as methylation (Me), phosphorylation (P), and acetylation (Ace), specialized enrichment techniques must often be deployed to obtain a sufficient ion count to detect and quantify the analyte of interest [32,33,34,35]. PTMScan^®^ is one such technique that takes advantage of PTM-specific (or PTM motif) antibodies that are bound to agarose or magnetic beads [36]. This technique effectively enriches the peptides that contain the modification and targets of interest. Nonetheless, such targets (including PTM-histones) can often be missed by occupying multiple charge states which dilute the signal intensity and display poor sorbent retentive properties. Indeed, histones that harbor many arginine and lysine residues are notoriously difficult to monitor through typical bottom-up LC-MS proteomics approaches, in that peptide products are often short hydrophilic species that cannot be easily separated through LC. Furthermore, the number of occurring modifications can force missed cleavage events which often go unaccounted for due to the logarithmic expansion of search libraries for multiple PTMs and/or localized PTM residues within a protein subdomain [37,38].

For the characterization of histone PTMs, the most common workflow is bottom-up proteomics with chemical derivatization. Nuclei are isolated using hypotonic buffers and histone proteins are extracted through sulfuric acid, precipitated with trichloroacetic acid (TCA), derivatized via propionylation, digested with trypsin, and analyzed through standard LC-MS setups using DDA or DIA methods [37]. Other workflows include the use of Glu-C digestion and/or upfront histone isolation strategies to identify and define crosstalk between co-occurring histone modifications [39]. To provide a suitable separation resolution for the modifications of interest, these strategies are often combined with weak cation exchange–hydrophilic interaction chromatography (WCX-HILIC) [40]. Here, we showcase the use of Affinity-Bead Assisted Mass Spectrometry (Affi-BAMS) as an orthogonal technique to LC-MS-based histone profiling. Specifically, we demonstrate that Affi-BAMS overcomes the need for LC separation or chemical derivatization of histone peptides and enables effective MALDI MS analyses while also providing for PTM-target multiplexing [41].

## 2. Results

**Figure 1:**
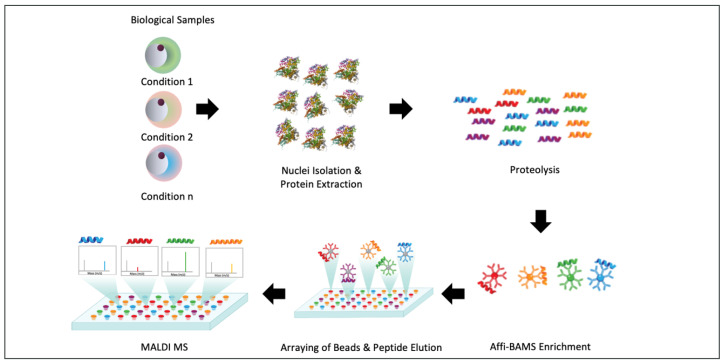
Affi-BAMS workflow for the measurement of histone PTMs. Samples are processed as isolated nuclei (or total cell lysates) to generate proteolyzed peptides. Target PTM-peptides are then enriched onto Affi-BAMS affinity beads and eluted. Eluted PTM-peptides are then measured by MALDI MS.

For the quantitative assay of PTM-modified histones, we first demonstrate a straightforward workflow that efficiently employs the digestion of isolated nuclei using standard bottom-up proteomics proteases and antibody-coupled magnetic beads to enrich histone-modified peptides with modifications of interest (Figure 1). Immunoaffinity beads with bound peptide analyte(s) are stringently washed to eliminate nonspecific binding and subsequently exposed to a MALDI matrix elution protocol that releases captured peptides into an array for analysis via MALDI MS. Our overall studies (1) define this platform’s technical performance using surrogate H3 and H4 peptides; (2) extend this workflow to cell line models; (3) highlight how specific modifications can be quantified; and (4) demonstrate how histones can be characterized for co-occurring PTM marks.

### 2.1. Optimization of MALDI Matrix Peptide Elution from Affinity Microbeads

For the MALDI MS component of our Affi-BAMS workflow, we first worked to benchmark the reproducible elution of peptides on MALDI MS-compatible microarrays. This specifically focused on optimizing several MALDI matrix components using an iMatrixSpray system. This was accomplished in a fume hood at a constant temperature (70 °F) at 50% humidity. Humidity was effectively maintained using a water-wicking tray, with hygrometer monitoring. For MALDI matrix application, the conditions assessed included the spray speed (mm/s), matrix density (uL/cm^2^), and uniformity achieved.

For sprayer needle parameters, the height was fixed at 70 mm, and the spray area was fixed at 80 × 40 mm. These values provided the full retention of beads in microwells, and full coverage of the Affi-BAMS array slide. We then optimized the spray flow liquid density and the spray speed based on the uniformity of the matrix deposition (Supplemental Figures S2–S6). A spray density of 5 µL/cm^2^ with 15 spray cycles was found to be optimal (Supplemental Figures S3 and S4). Spray densities of less than 5 µL/cm^2^, in contrast, did not maintain bead hydration over the entire elution cycle. For the spray speed, 60 mm/s was found to be optimal. Speeds of less than 50 mm/s generated nonuniform spots, while speeds of more than 100 mm/s overfilled array wells (which could lead to well cross-contamination) (Supplemental Figure S5). The line distance did not have substantial effects on the microarray quality (Supplemental Figure S2). Using the optimal settings of 5 uL/cm^2^ spray liquid, 60 mm/s speed, and 15 total spray cycles (at 1 mm line distance), uniform microarrays were generated that efficiently exposed microbeads to solvent for peptide elution. This work allowed uniform microarrays to be routinely generated with fully solvent-saturated beads to provide efficient peptide elution and crystallization in each/all wells (Supplemental Figure S6). The overall results are summarized in Table 1.

Next, we sought to investigate the spot acquisition parameters using a timsTOF Flex instrument. We first set up the geometry by designing a slide geometry file containing 88 × 26 spots. We next mapped the coordinates of the top left, top right, and bottom right spots in the microarray to align the geometry of the slide with the data collection. Through trial and error, we found that the M5 defocus, 150 × 150 µm beam scan using a random, partial sample with a spot size of 600 µm could be used to acquire data uniformly across the corresponding spot by visual inspection during laser discharge (Supplemental Figure S7A). To eliminate the background noise caused by matrix clusters, we assessed the addition of ammonium citrate to the elution matrix and acquired the resulting blank microarray using 20 and 40 percent laser power (Supplemental Figure S7B). Twenty percent laser power was more favorable, displaying a lower background signal, while having a minimum of 10 mM ammonium citrate completely negated the background noise at both tested laser power values.

### 2.2. Efficient Target Peptide Capture and Elution from Targeting Affinity Beads

We next advanced our study to confirm the reproducibility of target peptide capture, elution in microarrays, and MALDI MS analysis. Here, stable isotope peptides were used specifically as 3 picomoles of paired histone H4 surrogate peptides containing either a light or heavy lysine (K) isotope, such as SGRG[k(Ace)]GG[k(Ace)]GLG[k(+8)] (1185.67 and 1193.68 *m/z*). Peptides were incubated with triplicate Affi-BAMS beads, each of which contained a monoclonal antibody specific to the following four H4 acetylated lysines (Lys5, Lys8, Lys12, and Lys16) (shared epitope for these four H4 PTMs).

**Figure 2:**
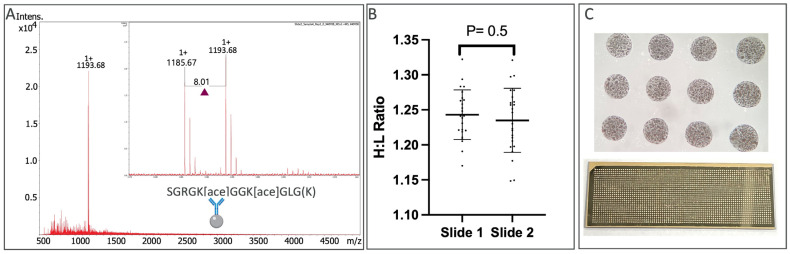
MALDI MS spectrum of the light and heavy histone H4 surrogate peptides, co-captured using the Affi-BAMS beads (Panel **A**). An unpaired *t*-test is displayed for H:L ratios of H4 surrogate peptide replicates across two arrays, with each replicate measurement depicted as a black dot (Panel **B**). Example of a reproducible microarray to the naked eye, and under 40× magnification (Panel **C**).

After overnight incubation at 4 °C, beads were washed and arrayed, and the resulting peptide microarray was printed using optimized iMatrixSpray conditions. To benchmark the variability among replicate samples for each assay in this workflow, H4 peptides were divided across 2 arrays with 8 samples and triplicate targeting beads employed (i.e., 24 beads per each two arrays). Clean signals were observed for both the light (1185.67 *m/z*) and heavy (1193.68 *m/z*) peptide surrogate samples with an expected +8 *m/z* mass shift of the heavy isotope labeled lysine derivative peptide (Figure 2A). Quantitative analysis of the ratio of heavy to light (H:L) indicated a coefficient of variation (cv) of 2.85% among replicates in array slide 1 and 3.70% for the replicates in slide 2. An unpaired t-test of the ratio of the means (1.24 and 1.23) showed no significant difference between the slides (*p* = 0.5) (Figure 2B). Direct observations showed reproducible features of each generated microarray (Figure 2C). Although the mean ratio between the two measured peptides was not exactly one, indicating a small discrepancy between the overall total amounts of peptides present in the sample (stemming from uneven yields of reconstituted lyophilized peptides), the high precision of quantification for the workflow was exemplified.

### 2.3. Linear Range of Sensitivity for the Assay of Histone H3 PTM-Peptides by Affi-BAMS

**Figure 3:**
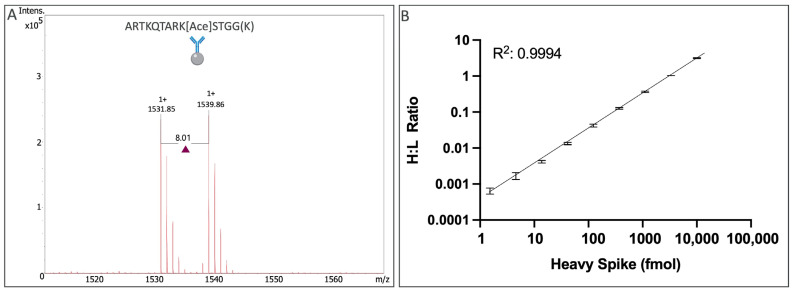
MALDI mass spectrum of on-bead immunoaffinity peptide enriched histone H3 peptide surrogates at an equimolar spike of 3 picomoles (Panel **A**). Intensities of the light and heavy peptides were recorded and the ratio was plotted at each corresponding heavy spiked peptide quantity (Panel **B**).

To further define the platform’s performance, we next assessed quantitative measurements and assay range using histone H3 surrogate peptides to focus on platform and workflow components. Histone H3 surrogate peptides with the following light or heavy lysine (K) isotope sequences were used: ARTKQTAR[k(Ace)]STGG[k(+8)]. The heavy lysine isotope H3 peptide was spiked into a Tris-based buffer at varying total amounts (1.52 femtomole—10 picomoles), while the light lysine isotope H3 peptide was spiked at a fixed total peptide level of 3 picomoles. To capture these target peptides, an H3 acetyl-Lys9 immunoaffinity bead was used. Enrichment was performed overnight using three replicate beads per peptide dilution point. For light (1531.85 *m/z*) and heavy (1539.86 *m/z*) peptides, clean signals were acquired (Figure 3A). A linear correlation of signals was observed for the H:L ratio across the dilution range of approximately three orders of magnitude, with an R2 value of 0.9994 (Figure 3B).

### 2.4. Effects of Anionic Detergents on Immuno-Affinity Bead Peptide Capture

**Figure 4:**
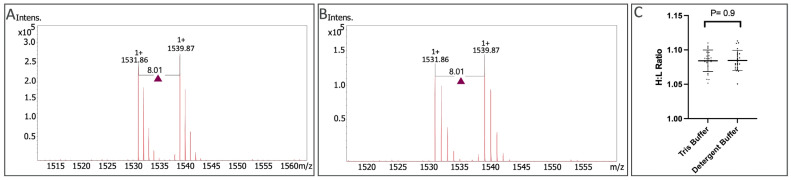
MALDI mass spectra of on-bead immunoaffinity enriched histone H3 peptide surrogates at equimolar spikes (3 picomoles) in Tris-based buffer (Panel **A**) and detergent-based buffer (Panel **B**). Intensity values for the heavy and light peptides were extracted, and the ratios were calculated as H:L; an unpaired *t*-test was calculated for the replicates, with each replicate measurement depicted as a black dot (Panel **C**).

We next assessed the robustness of target capture by affinity beads in the presence of ionic detergents commonly used in MS sample workflows [42]. Specifically, a Tris buffer system (100 mM KCL, 100 mM Tris, pH 7.5) and a detergent buffer system (0.5% Sodium deoxycholate, 12 mM sodium lauryl sulfate, 50 mM ammonium bicarbonate) were assessed. Light and heavy lysine isotope H3 peptides were spiked into each buffer at 3 picomoles per sample (28.57 femtomole/uL), including eight replicates for each condition. Histone H3 Acetyl-Lys9 affinity beads were then used to capture H3 peptides (with triplicate beads used for each sample). Sharp signals were observed for light (1531.86 *m/z*) and heavy (1539.87 *m/z*) lysine isotope H3 peptide samples for the Tris-based buffer (Figure 4A) and for the detergent-based buffer (Figure 4B). An unpaired *t*-test of H:L ratios showed no significant difference between the groups (*p* = 0.9), or between the coefficient of variation of replicates with (1.36% cv) or without (1.45% cv) detergent (Figure 4C).

### 2.5. Multiplexed Histone H3 and H4 PTM-Peptide Enrichment and MALDI Analysis Using Nuclear Lysates

**Figure 5:**
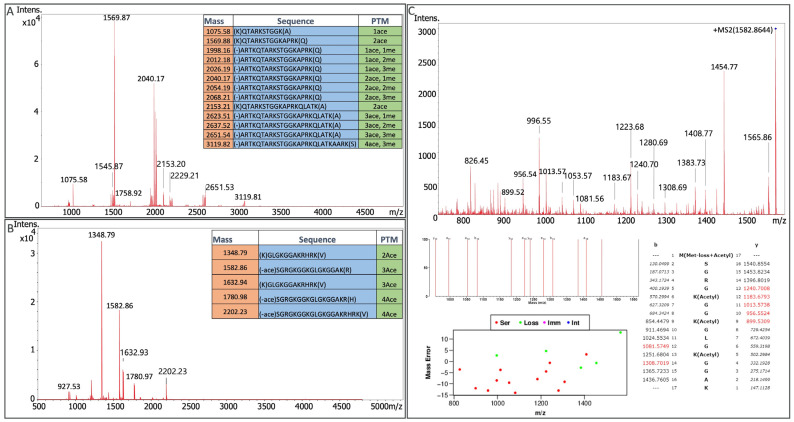
MALDI mass spectra of histone H3 (Panel **A**) and histone H4 (Panel **B**) peptides enriched via immunoaffinity captured from HeLa nuclei lysate using Affi-BAMS beads. Fragmentation of the histone H4 peptide with three acetylation modifications and y and b fragment ion matching in ProteinProspector (Panel **C**).

Analyses of histone H3 and H4 PTMs were next performed using HeLa cells as nuclear lysates. Nuclei isolation enriches nuclear proteins (and decreases protease requirements). Using trypsin-digested nuclei from 1 million HeLa cells, enrichments were conducted for H3K9ace and H4K5, 8, 12, and 16ace target peptides. The analyses defined a unique set of peaks for each immunoaffinity bead (Figure 5A,B). For histone H3K9ace, in addition to the anticipated peptide at 1075.58 *m/z* that corresponds to the sequence (K)QTAR[Kace]STGGK(A), twelve additional peaks were observed that corresponded to distinct acetyl modifications and mono-, di-, and trimethylations at amino acids proximal to lysine 9 of histone H3 (Figure 5A). For histone H4, we similarly observed peptides with *m/z* values matching H4 peptides with two to four acetylation modifications (Figure 5B). Notably, these findings demonstrate that histone targeting beads can be enriched for combinatorial histone modifications adjacent to a specific modification of interest.

To confirm peptide identities, we subjected enriched histone H4 peptide to collision-induced dissociation fragmentation (CID). Fragment ions were then searched through the MS-Tag ProteinProspector software module [43] with a histone H4 user protein sequence (Figure 5C). We were able to observe and define thirteen H3 proteoforms and five H4 proteoforms within a single multiplexed Affi-BAMS experiment. These observed analytes are summarized in Table 2.

**Figure 6:**
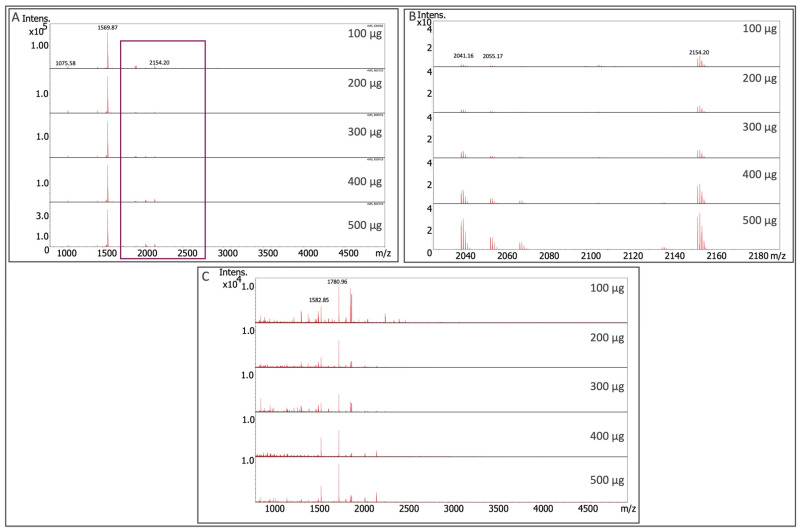
MALDI mass spectra of signals recovered from varying LysC digested soluble protein input materials utilizing the histone H3 (Panel **A** and **B**) and histone H4 (Panel **C**) Affi-BAMS beads from HCT116, COLO 205, and MOLP-8 nuclei lysates. Although Panel A shows 1569.87 *m/z* as the most intense histone PTM analyte, Panel B highlights the lower intensity analytes from the same spectra files attributed to several other histone peptides co-captured via enrichment.

To assess the protein input requirements from nuclei lysates, a mixture of cell lines was utilized to create a combined stock lysate which included HCT116, COLO 205, and MOLP-8 cells. This was aimed at generating a mixture of lysates containing several combinatorial H3 PTMs. Multiple H3 peptides were observed with dually acetylated 1569.87 *m/z*, QTARK(Ace)STGGK(Ace)APRK as the predominant MS signal (Figure 6A). Magnification of the boxed spectra region further shows several H3 proteoforms with an overall lower signal-to-noise ratio (Figure 6B). For histone H4, several acetylated peptides were observed with 1780.96 *m/z*, SGRGK(Ace)GGK(Ace)GLGK(Ace)GGAK(Ace)R as the peak signal (Figure 6C). For nuclear lysates, this illustrates the effective use of a protein sample of as little as 100 μg.

### 2.6. SILAC and HDAC Inhibitor-Based Assessment of Histone H3 via Affi-BAMS

**Figure 7:**
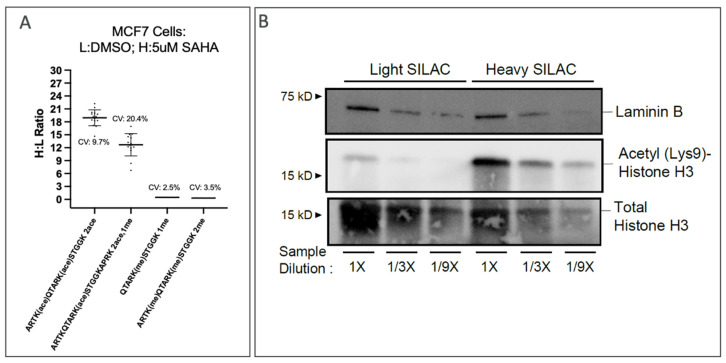
Heavy to light (H:L) ratio of analytes identified from MALDI mass spectra recovered from histone H3K9ace and H3K9me Affi-BAMS beads from MCF7 cells treated with 5 uM SAHA for 24 h (Panel **A**). The Western blot analysis confirmed the upregulation of H3K9 acetylation in MCF7 cells treated with 5 uM SAHA (Panel **B**).

In experiments aimed at further defining precision and quantitative measures of Affi-BAMS, SILAC labeling studies were performed. In addition, the HDAC inhibitor SAHA was included (+/−5 uM, 24 h) in this design. Specifically, we cultured MCF7 cells in light and heavy (R + 10 and K + 8) SILAC media, where the heavy proteome was treated with 5 uM SAHA for 24 h, while the light control was treated with DMSO. The heavy-to-light ratios were generated for each analyte and plotted (Figure 7A and Supplemental Figure S9). The combined analytical and biological reproducibility was observed to be <10% (CV) on average among the measured analytes. Importantly, the expected outcome of increased histone H3 acetylation upon HDAC inhibition was observed. To confirm these results, we performed Western blot analysis probing for H3K9ace along with laminin B and total histone H3 as loading controls (Figure 7B).

Notably, when treating MCF7 cells with an HDACi (5 uM SAHA), we observed a significant increase in acetylation levels on H3K9ace, along with one additional acetyl modification (two acetyls in total). Given that the addition of a modification would force a missed cleavage event and only two additional lysine residues were unmodified, the additional acetylation modification is indicated to be located on H3K4 (ARTKace). Similarly, we considered that the additional methyl modification found on the two methyl-modified peptides on H3K4 (ARTKme) is likely to be due to a missed cleavage. When two acetylation modifications and one methylation modification are observed, it is difficult to assign the localizations of the modifications (due to multiple possibilities).

## 3. Discussion

In these studies, we employed a unique immunoaffinity bead and MALDI MS platform to enable targeted assays for PTM-modulated histones reported on both targeted and proximal histone marks. We reported on the assessment of the technical components of the Affi-BAMS platform, including the (1) elution process of bead-captured-peptides arrayed on microarray slides and (2) confirm the reproducibility of the signals of peptides captured using H3 and H4 PTM peptides. We further assessed and confirmed the compatibility of peptide retrieval in the presence of ionic detergents which had minimal effects on the quantification precision of the assay. Using H3 and H4 surrogate PTM peptides, we demonstrated the dynamic range and reproducibility achievable with the assay, both within and between microarrays. To test and determine the effectiveness of the assays, we extended the studies to human cell lines and were able to identify the vast heterogeneity of histone PTMs afforded by the antibody reagent. By incorporating SILAC labeling, we demonstrated the quantification of histone PTMs using a well-established histone deacetylase inhibitor.

Proteomic bead-based assays, with respect to multiplexed immuno-affinity analysis, rely primarily on fluorescence-based detection methods, which are not able to provide the level of detail required to fully resolve the diverse composition of captured analytes afforded by the reagent antibody, such as in the cases of the proximity extension assay (PEA) or ELISA-based assays. In contrast, Affi-BAMS provides detection via MALDI MS, which produces a high-resolution, molecular characterization of the captured analytes. Exemplified by our MCF7 SILAC-based HDAC inhibition using SAHA, the upregulation of acetylation on H3K9 is clear. However, with Western blot, it is unclear what other modifications are unaccounted for under this single fluorescing band. Using Affi-BAMS, we resolved that this upregulation is primarily accounted for by two acetylation marks that are also associated with a methyl mark on the same peptide region. MALDI MS detection maximizes the information content afforded by the antibody reagent by providing a comprehensive inventory of captured peptides with the target epitope with the high on-bead multiplexing capacity for each Affi-BAMS bead capture. In addition, the microarray format of the Affi-BAMS workflow allows one to independently probe many analytes by using different Affi-BAMS beads within the same multiplexed experiment. Since each Affi-BAMS bead is isolated into a dedicated microwell, the multiplex assay can probe many different types of biomarker peptides to monitor the total protein content, multiple different post-translational modifications on a single or different proteins, combinatorial PTMs within a local region of a particular protein to survey proteoforms, or even the accumulation of specific point mutations on a single protein or multiple proteins [41]. Due to this unique level of multiplexing, the Affi-BAMS platform can be effectively used as an efficient biomarker screening workflow. These attributes position the platform as a scalable quantitative method when screening for disease biomarkers, stratifying patients within a particular disease group, or monitoring point mutations that frequently arise to confer drug resistance. Additionally, Affi-BAMS holds potential as a rapid analytical tool within the drug design process. It can be integrated into a biochemical enzyme assay, advanced to a cell-based assay, and ultimately utilized as an efficacy marker readout. The platform’s versatility enables the efficient analysis of proteins, peptides, and PTMs, making it an invaluable tool.

We have demonstrated the use of Affi-BAMS for a variety of applications as a targeted bioanalytical tool for understanding cellular signaling in this work and previous work [41]. These mechanisms, although complex, can be dissected by focusing on specific protein targets of interest. Our previous studies show how multiple PTM sites can be resolved, quantified, and localized on single signaling proteins, such as *RPS6*, *ERK1/2*, *AKT1/2/3*, and *STATs* [41]. In this study, we demonstrate how the Affi-BAMS assay can be easily adapted by modifying the sample preparation methods and Affi-BAMS reagents to gain deeper insights into PTMs or protein processing events that are found on histone proteins.

We recognize that this technology requires an intact epitope for antibody recognition. One can mitigate this challenge by utilizing alternative proteases that produce different peptides by cleaving at diverse residues that flank the epitope region. The use of different proteases will provide complementary information to further characterize the targeted PTM sites of interest, or to reveal new PTMs that occur upstream or downstream of the epitope region while being contained within a larger proteolytic peptide fragment. We demonstrate this through histone H4 acetyl peptides generated from LysC and ArgC protease digestion of HeLa nuclei lysate (Supplemental Figure S8). This occurs when using proteases with distinct cleavage sites to generate longer fragments that maintain the combinatorial histone PTMs on a single peptide product.

A limitation to deciphering the stoichiometry of a given histone PTM in reference to the unmodified counterpart is inherent to PTM-directed antibody enrichment, as the unmodified proteoform will not be enriched and therefore quantified. Fortunately, users can utilize an upstream epitope-directed antibody to enrich both forms (modified and unmodified) of interest. Epitope exclusion is another limitation that is inherent to antibody-based platforms. While Affi-BAMS may be subjected to this pitfall, the platform has an inherent advantage over other antibody-based analytical platforms. Having MALDI MS as the analytical measurement overcomes ambiguity as to the origin of the peptide intensity and avoids interference from the cross-reactivity of poorly selective antibodies. Potential limitations are to localize secondary modifications that are co-occurring and co-captured during the enrichment process. Because the platform provides MS1-based quantification and identification, localization information is often not explicit due to the isobaric nature of certain peptides. Fortunately, users can carry out MS/MS sequencing and correlate the fragmentation patterns to localize the modification of interest and thus infer the identity back to the intact peptide analyte quantified from the same spot (or location) within the microarray. Alternatively, Affi-BAMS beads for a given target can be isolated (Eppendorf tube format), and the captured peptides can be eluted for subsequent ESI-LC-MS/MS analysis for the sequence identification and localization of corresponding PTMs.

The utility of the platform to understand the selectivity of biopharmaceutical agents in development and to uncover relationships between co-occurring histone modifications upon treatment is clear. Future work will aim to develop Affi-BAMS assays to obtain global coverage of PTMs found on histone proteins.

## 4. Materials and Methods

Affi-BAMS Reagents: Adeptrix Corp (Beverly, MA, USA), Cat # R0304, R0312, and R0305.
Affi-BAMS Matrix Sprayer: Adeptrix Corp (Beverly, MA, USA), Cat # C0005.
Affi-BAMS Assay Kit: Adeptrix Corp (Beverly, MA, USA), Cat # C0008.
Affi-BAMS Microwell Array: Adeptrix Corp (Beverly, MA, USA), Cat # C0011.

### 4.1. Sample Generation

Cells were cultured in DMEM or RPMI media with 10% fetal bovine serum (FBS) and 1X Pen-Strep (Sigma, Burlington, MA, USA, #P4333) to 75% confluence at 37 °C with 5% CO_2_. Treatment was conducted as indicated in the results with matched timepoint DMSO controls. SILAC-labeled cells were grown in either normal arginine and lysine or heavy arginine (+10) and lysine (+8) (ThermoFisher Scientific, Waltham, MA, USA, Cat #A33972) for at least six passages. Cells were washed twice with Phosphate Buffered Saline (PBS) before cells were pelleted and snap-frozen. SILAC conditions were harvested during the exponential phase. The SILAC heavy MCF7 cells were treated with 5 uM HDACi Vorinostat for 24 h (SAHA, Millipore Sigma, Burlington, MA, USA, Cat # SML0061), while the light SILAC was treated with DMSO.

### 4.2. Sample Preparation

Nuclei were isolated from each cell pellet by the addition of 15 mM Tris-HCl, 60 mM KCl, 15 mM NaCl, 5 mM MgCl_2_, 1 mM CaCl_2_, 250 mM sucrose, 0.3% NP40, 1 mM DTT, 10 mM Na-butyrate, and incubation on ice for 5 min followed by centrifugation. NP40 was removed by washing with 15 mM Tris-HCl, 60 mM KCl, 15 mM NaCl, 5 mM MgCl_2_, 1 mM CaCl_2_, and 250 mM sucrose.

Lysis was performed via the addition of 0.2% RapiGest^TM^ SF (Waters Corp, Milford, MA, USA, Cat # 86001861) in 100 mM ABC directly to isolated nuclei. Protein concentration was performed using the Pierce™ BCA Protein Assay (ThermoFisher Scientific, Cat # 23225) following the manufacturer’s protocol. LysC digestion (ThermoFisher Scientific, Waltham, MA, USA, Cat # 90051) was conducted using a 1:50 enzyme: protein ratio and incubated overnight at 37 °C. Samples were boiled to denature protease prior to proceeding to enrichment.

Peptide enrichment was typically performed with 3 replicate beads per target per sample following the manufacturer’s protocol. Peptides and affinity capture beads were incubated for a period of 12 h at 4 °C in a Thermomixer (Eppendorf, Hamburg, Germany). Beads were subsequently washed sequentially in 1 M KCl, 100 mM Tris HCl (700 µL, 10 min), 10 mM ammonium bicarbonate (700 µL, 2 min) twice, and deionized water (700 µL, 2 min) twice at 4 °C. Beads were transferred between individual washes using a QuicPick magnetic bead handler (Bio-Nobile, Cat # 24001).

The washed Affi-BAMS beads were carefully transferred to the hydrated wells of the microarray. The beads settled into the microwells with gentle agitation; the sample chamber gasket was removed, leaving the microwell gasket fixed in place on the slide. Residual water was removed by padding on a dry towel with a magnet placed on the opposite side to hold the Affi-BAMS beads in place within the microwells.

The bead-captured peptide was eluted off using a matrix sprayer containing 0.5 mg/mL α-cyano-4-hydroxycinnamic acid (CHCA) in 50% acetonitrile and 0.5% trifluoroacetic acid (TFA). The matrix sprayer (iMatrixSpray) was obtained from Tardo GmbH (Subingen, Switzerland). The aerosol was applied to the surface of the array with the microwell gasket and beads facing the MALDI matrix sprayer. Through manual refinement, the iMatrixSpray parameters were set to the following: height, 70 mm; line distance, 1mm; speed, 60 mm/s; density, 5 µL/cm^2^; number of cycles, 15; delay, 0 s; spray area width, 80 mm; and spray area depth, 40 mm. This provided reproducible microarrays given a humidity of 50% and stable temperature of around 70 °F.

Post-elution cycle, residual solvent on the microarray evaporated, leaving crystalized peptide targets within each microarray spot. The silicone gasket was removed, and beads were displaced using compressed air. Prior to MS data acquisition, analyte-containing spots within each microarray were identified by visual inspection. The analyte-containing spots were identified based on their characteristic appearance due to the presence of an inner area devoid of the MALDI matrix.

### 4.3. Mass Spectrometry Analysis

MALDI TOF MS data were acquired with the Bruker Daltonics (Billerica, MA, USA) timsTOF fleX instrument using timsControl to collect the precursor mass signal. The microarray geometry file was set to contain 88 × 26 spots across the microarray, and a teaching schema was generated to fit the array. Mass spectra were acquired in positive linear mode in the 850–5000 *m/z* mass range using 20–40% laser power and 2000 laser shots with a laser frequency of 10,000 Hz per spot. The laser application was set to custom mode (M5 defocus) with a 600 um spot size with the walk-on-spot mode set to the random partial sample. In the case of MS/MS sequencing, the instrument was set to collect MS/MS with several different collision energy values to obtain sufficient fragmentation patterns for the quadrupole-selected precursor.

Mass spectra were processed and analyzed using Compass DataAnalysis software. Unless otherwise indicated, the software algorithm labeled peaks using either average or monoisotopic *m/z* values.

Peptide sequencing data were analyzed using MS-Tag (ProteinProspector, University of California San Francisco). The MS-Tag settings were as follows: database, user-defined protein sequence for histones H3 and H4; taxonomy, Homo sapiens; digest, no enzyme; variable mods, acetyl (K), deamidated (R), methyl (K), dimethyl (K), trimethyl (uncleaved K), phospho (ST), Gln->pyro-Glu (N-terminal Q), and oxidation (M); parent ion tolerance, 10 ppm; fragment tolerance, 10 ppm; max mods, 4; and instrument, MALDI-TOFTOF.

## Figures and Tables

**Table 1:** Parameters assessed on the iMatrixSpray sprayer to create uniform microarrays; optimal settings are marked with an asterisk and bolded.

Components Tested	
Spray Speed (mm/s)	30, 50, 60 *, 70, 90, 110, 130
Height (mm)	30, 40, 50, 60, 70 *
Spray Density (uL/cm^2^)	1, 3, 5 *
Spray Cycle	5, 10, 15 *
Line Distance (mm)	1, 2

**Table 2:** A list of analytes identified from the immunoaffinity enrichment highlighted in Figure 5.

Protein	Protease	Sequence	Expected *m/z*	PTM
Histone H3	LysC	QTARKSTGGK	1075.58	1Ace
Histone H3	LysC	ARTKQTARKSTGGK	1545.88	1Ace1Me1
Histone H3	LysC	QTARKSTGGKAPRK	1569.88	2Ace
Histone H3	LysC	ARTKQTARKSTGGKAPRK	2040.18	2Ace1Me1
Histone H3	LysC	ARTKQTARKSTGGKAPRK	2054.19	2Ace1Me2
Histone H3	LysC	ARTKQTARKSTGGKAPRK	2068.21	2Ace1Me3
Histone H3	LysC	QTARKSTGGKAPRKQLATK	2153.21	3Ace
Histone H3	LysC	QTARKSTGGKAPRKQLATKAARK	2621.5	4Ace
Histone H3	LysC	ARTKQTARKSTGGKAPRKQLATK	2637.52	3Ace1Me2
Histone H3	LysC	ARTKQTARKSTGGKAPRKQLATK	2651.54	3Ace1Me3
Histone H3	LysC	ARTKQTARKSTGGKAPRKQLATKAARK	3091.79	4Ace1Me1
Histone H3	LysC	ARTKQTARKSTGGKAPRKQLATKAARK	3105.81	4Ace1Me2
Histone H3	LysC	ARTKQTARKSTGGKAPRKQLATKAARK	3119.82	4Ace1Me3
Histone H3	LysC	ARTKQTARKSTGGKAPRKQLATKAARK	3119.78	5Ace
Histone H4	LysC	GLGKGGAKRHRK	1348.79	2Ace
Histone H4	LysC	SGRGKGGKGLGKGGAK	1582.87	3Ace
Histone H4	LysC	GGKGLGKGGAKRHRK	1632.94	3Ace
Histone H4	LysC	SGRGKGGKGLGKGGAKR	1780.98	4Ace
Histone H4	LysC	SGRGKGGKGLGKGGAKRHRK	2202.23	4Ace

## Data Availability

Data can be found in the Supplementary Material.

## Supplementary Materials

The supporting information can be downloaded at: https://www.mdpi.com/article/10.3390/ijms241210060/s1.
