# Supplementary material for: Affi-BAMS™: A Robust Targeted Proteomics Microarray Platform to Measure Histone Post-Translational Modifications
Source: Int J Mol Sci. 2023 Jun 13;24(12):10060. doi: 10.3390/ijms241210060 (PMC10298961; doi:10.3390/ijms241210060)
Supplement: Supplementary file 1 [file ijms-24-10060-s001.zip › ijms-2444143-supplementary.pdf]

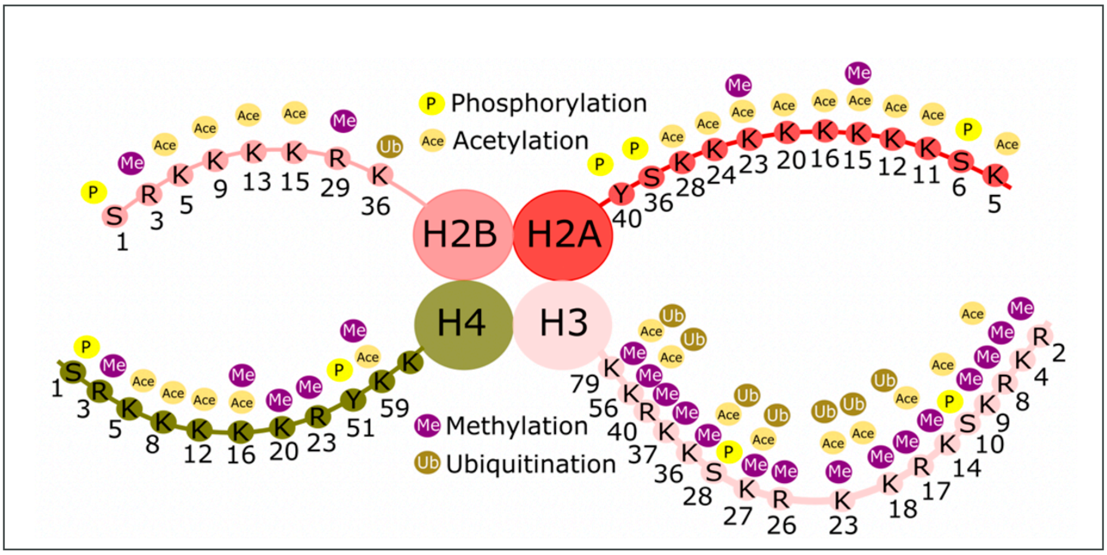

Supplemental Figure S1. A simplified modification landscape of Histones H2A, H2B, H3 and H4 octamer.

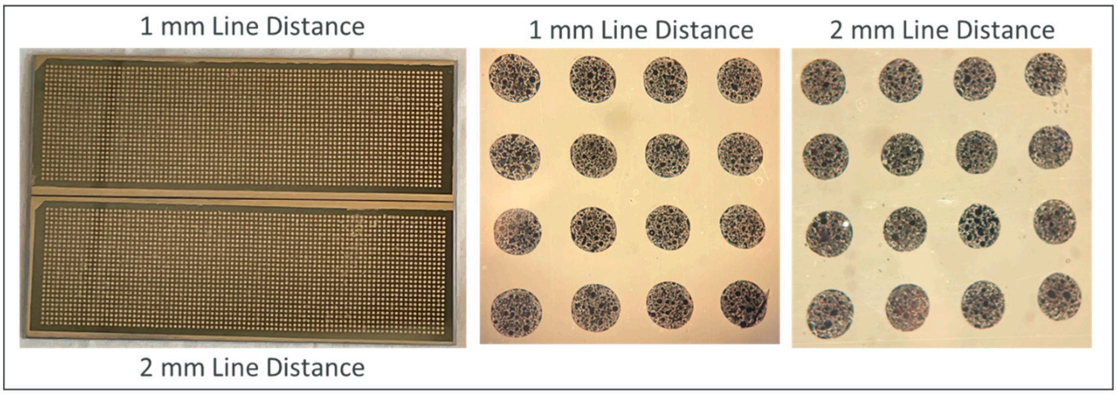

Supplemental Figure S2. Line distance of 1mm and 2mm do not show change in spot uniformity or the quality of the matrix crystallization under 40x magnification.

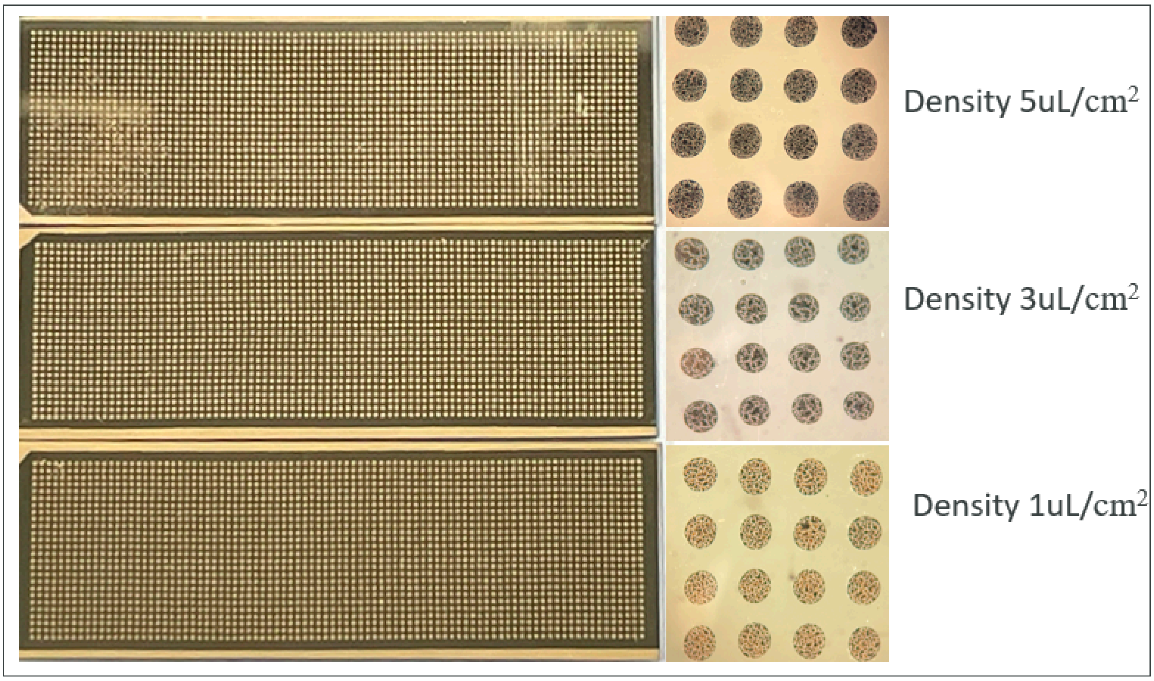

Supplemental Figure S3. The spray density was tested by varying the amount of solvent sprayed per second (1, 3 and 5uL/cm^2). The quality of the arrays was unchanged to the naked eye, while under 40x magnification, showed the greatest uniformity under 5uL/seconds.

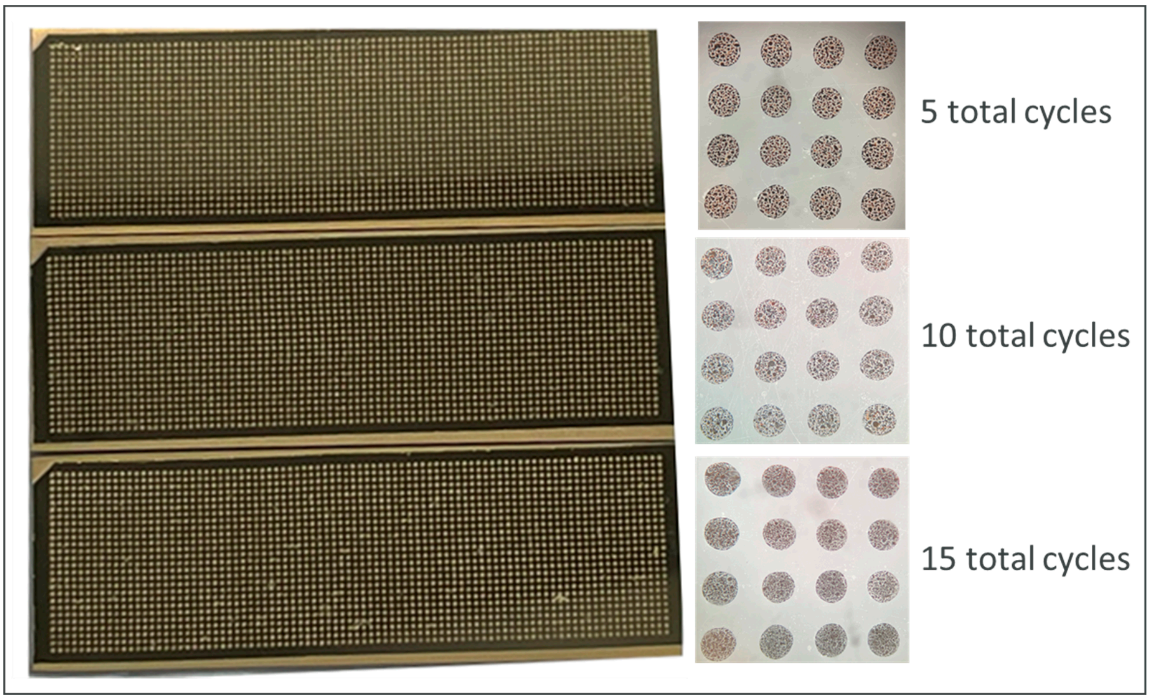

Supplemental Figure S4. The spray cycle was tested by varying the number of total cycles that the array is exposed to (5, 10 and 15 rounds of elution). The quality of the arrays was unchanged to the naked eye, while under 40x magnification, 15 total cycles displayed the most uniform and matrix filled arrays.

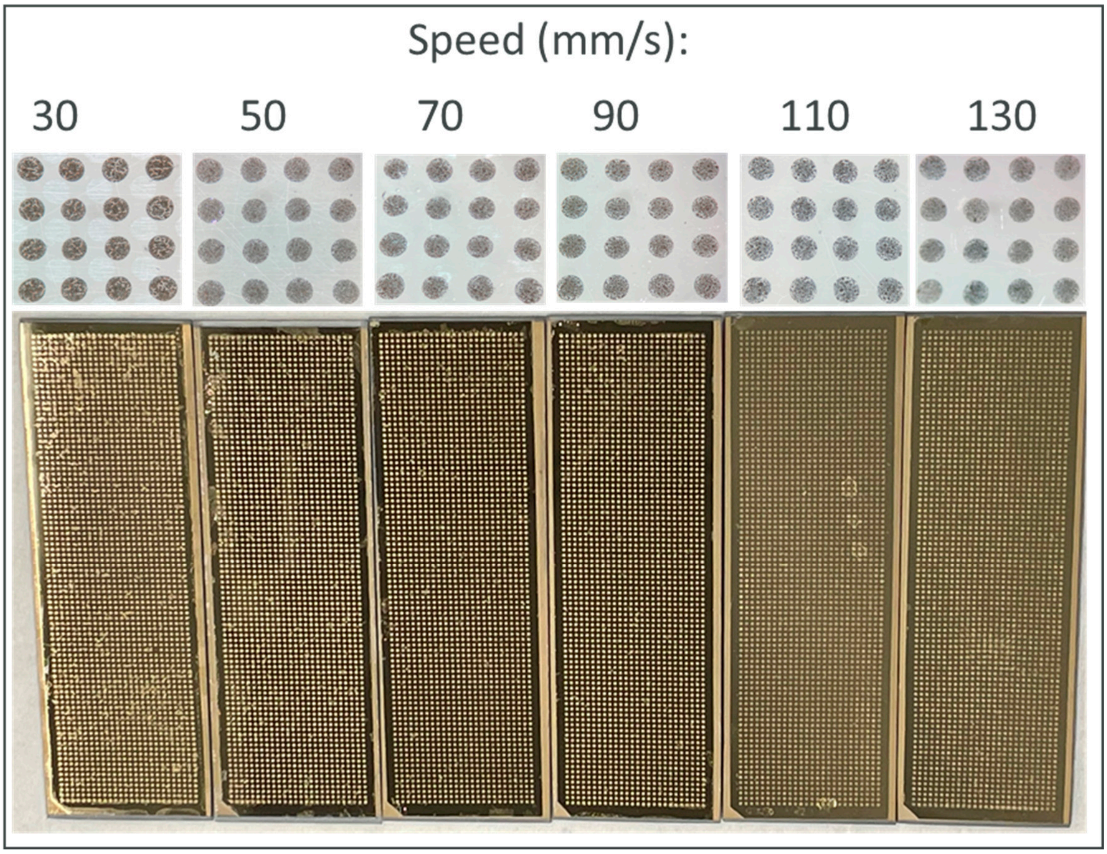

Supplemental Figure S5. The spray speed was tested by varying the speed of which elution needle moves across the microarray (mm/s). Of the six speeds tested, no obvious differences were seen under 40x magnification post elution process, however, to the naked eye speeds above 100 displayed excess solvent moisture leading to cross contamination from well-to-well.

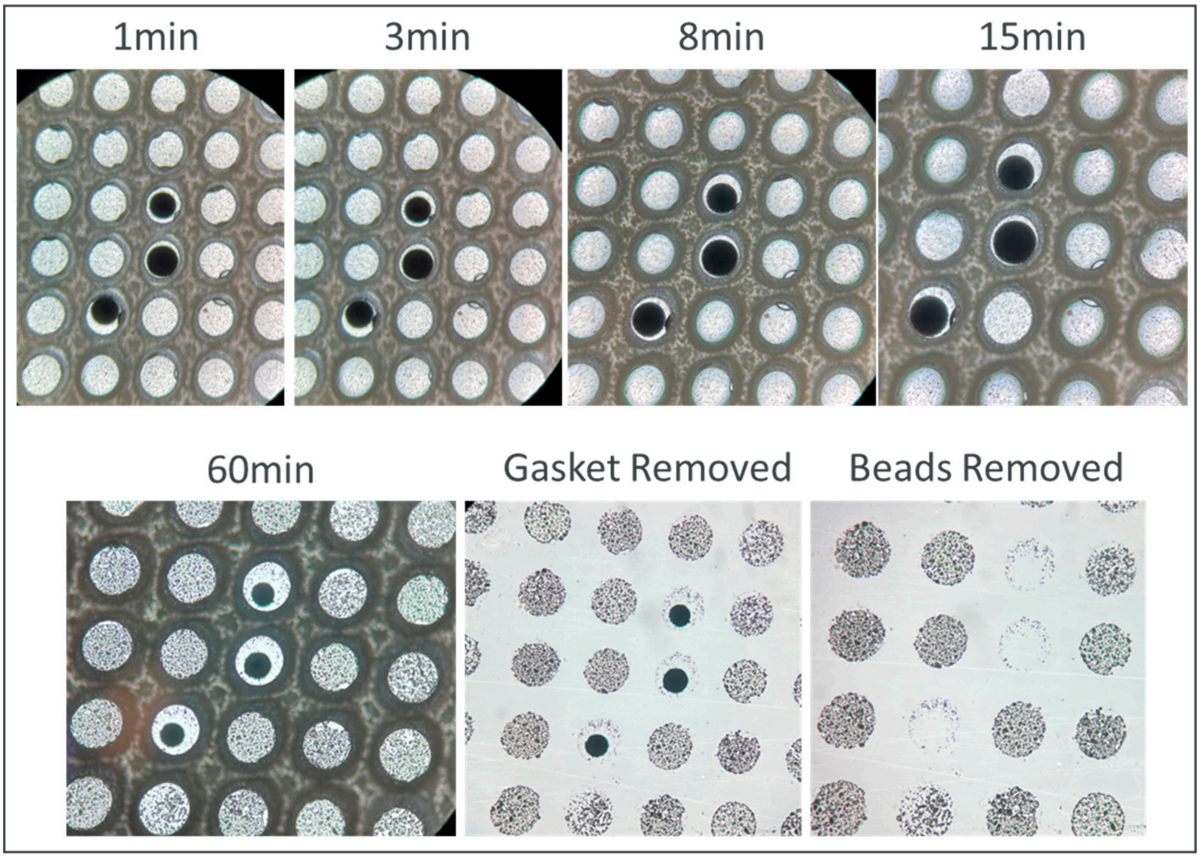

Supplemental Figure S6. Using the optimal settings identified, three beads were exposed to the elution process and images were captured under 40x magnification of a time-lapse of the drying process for 1, 3- , 8-, 15- and 60-minutes post elution. The gasket is removed while the beads are affixed in place and are subsequently removed via compressed air.

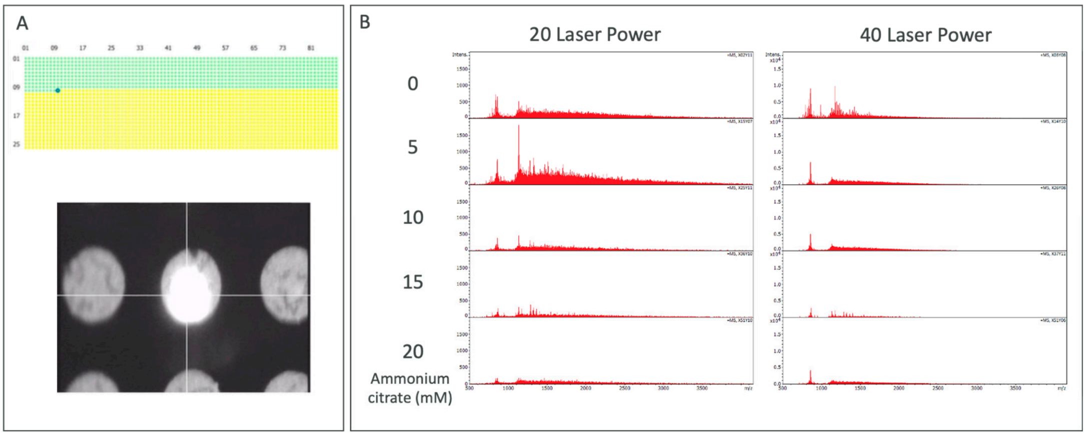

Supplemental Figure S7. An 88x26 spot microarray geometry file was implemented on the timsTOF flex instrument to match the generated microarray slide (Panel A). Parameters were selected to obtain uniform acquisition of the entire spot during laser discharge. A series of concentrations of Ammonium citrate was assessed to eliminate background noise from matrix adducts at 20 and 40 percent laser power (Panel B).

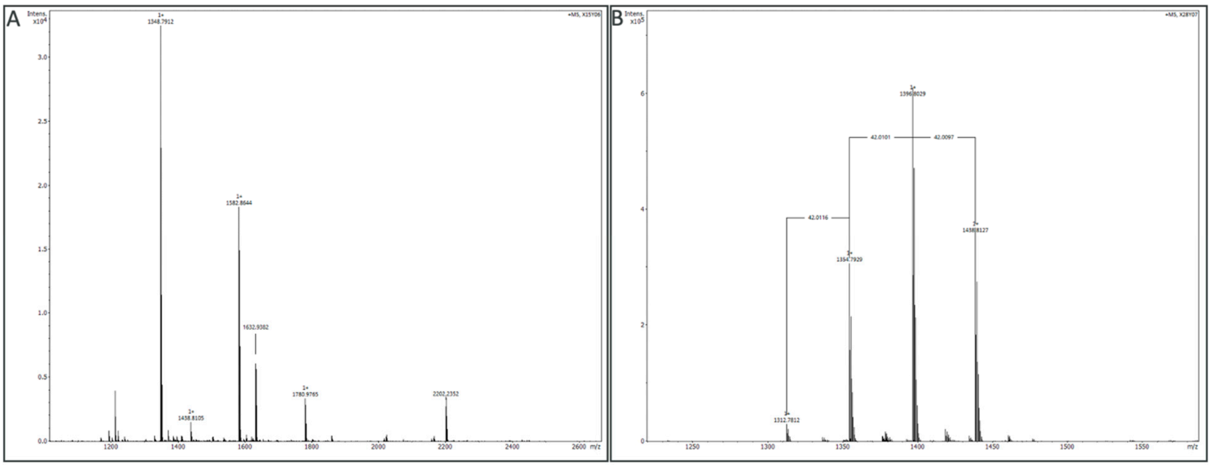

Supplemental Figure S8. MALDI mass spectra of Histone H4 immunoaffinity peptides from a LysC protease digest (Panel A) and ArgC protease digest (Panel B) captured from HeLa nuclei lysate using Affi-BAMS beads.

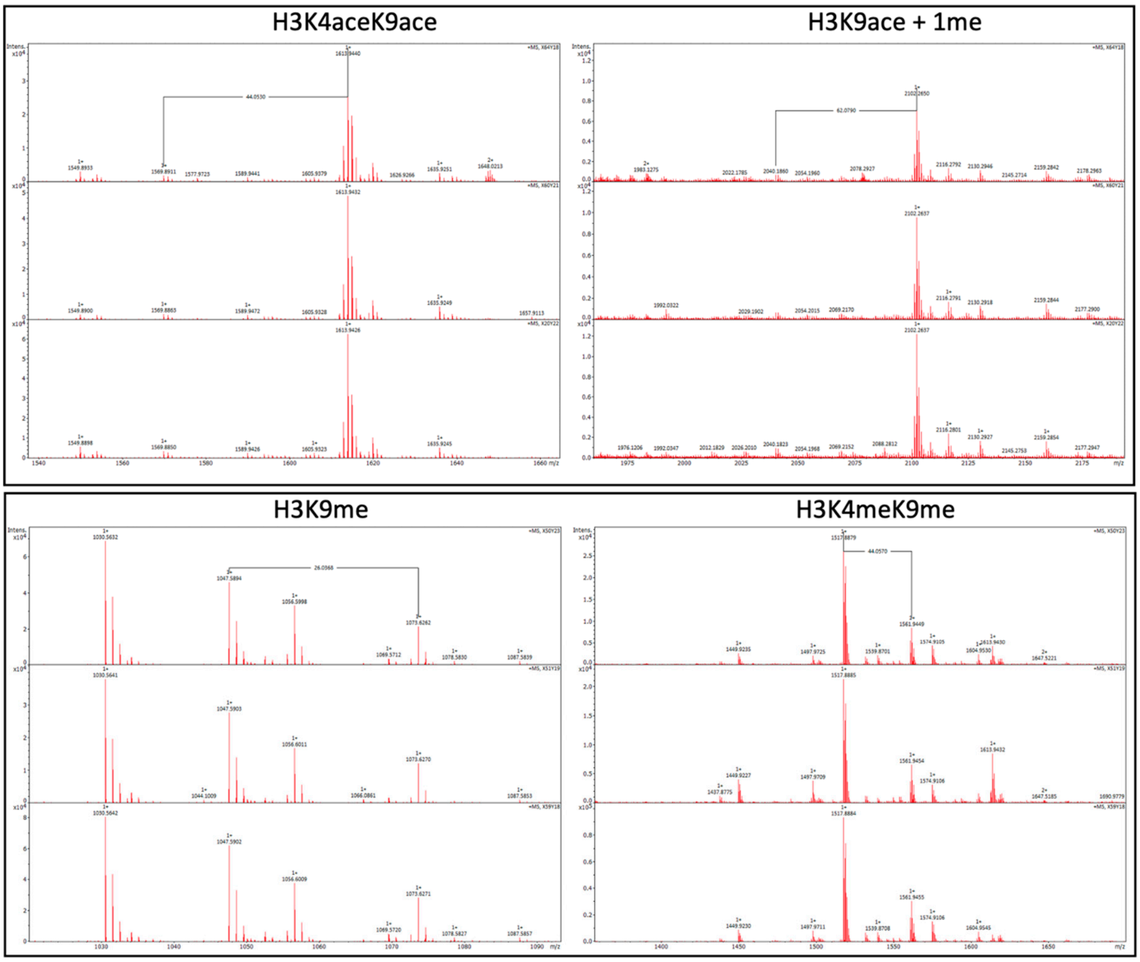

Supplemental Figure S9. MALDI mass spectra examples of Histone H3 immunoaffinity peptides from a LysC protease digest captured from MCF7 SILAC labeled nuclei lysate using Affi-BAMS beads.
